# Pulmonary Arterial Hypertension as a Fatal Complication of Neurofibromatosis Type 1 in a Neonate: A Diagnostic Dilemma

**DOI:** 10.7759/cureus.17137

**Published:** 2021-08-12

**Authors:** Sinan Yavuz, Mona K Abdullah, Nader Francis

**Affiliations:** 1 Department of Pediatrics/ Specialist Pediatric, Al Qassimi Women and Children Hospital, Sharjah, ARE; 2 Department of Pediatrics /Consultant Pediatrics and Neonatology, Al Qassimi Women and Children Hospital, Sharjah, ARE; 3 Department of Pediatrics/ Consultant Pediatric Pulmonology, Al Qassimi Women and Children Hospital, Sharjah, ARE

**Keywords:** neurofibromatosis type 1 (nf-1), idiopathic pulmonary arterial hypertension, genetic syndromes, congenital disease, preterm neonate

## Abstract

Neurofibromatosis type 1 (NF1), or von Recklinghausen disease, is a genetically transmitted autosomal dominant disease, with a prevalence of one per 4000 live births. Pulmonary arterial hypertension (PAH) is a rare but potentially life-threatening complication of NF1. There are no confirmatory data about the congenital association between PAH and NF1. However, in most cases, PAH is observed in late childhood or adulthood. Herein, we present a preterm baby with genetically confirmed NF1 who presented with PAH.

## Introduction

Neurofibromatosis type 1 (NF1), or von Recklinghausen disease, is an autosomal dominant multisystem disease, with a prevalence of approximately one per 4000 live births. It is characterized by café au lait macules, axillary and inguinal freckling, and benign neurofibromas with Lisch nodules. Further, the other manifestations are cardiovascular, gastrointestinal, renal, and endocrine system abnormalities and peripheral and central nervous system malignancies [1].

Due to defects in circulatory system adaptation at birth, resulting in persistently high pulmonary vascular resistance, pulmonary arterial hypertension (PAH) is considered a life-threatening disease among newborns [2]. The essential treatment is inhaled nitric oxide, which improves oxygenation without causing systemic hypotension, with an incidence of only two per 1000 live births [3].

Diffuse lung involvement can be observed in NF1 [4,5]. Based on the recently updated classification, PAH associated with neurofibromatosis with unclear and/or multifactorial mechanisms is classified under group 5 [6]. However, there are no clear data about the association between PAH and neurofibromatosis in the neonatal age group.

Herein, we report a preterm baby who was born at 27 weeks, four days gestational age. He was admitted to the neonatal intensive care unit after delivery because of respiratory distress. With consideration of clinical, radiological, and echocardiography findings, treatment failure, and a previous history of three abortions of unknown causes, the baby was suspected of congenital lung developmental disorders. Therefore, we decided to send whole-exome sequencing (WES), which showed the homozygous *NF1* (NM_000267.3) variant (c.7127-1=/G>T P).

## Case presentation

The preterm male baby was delivered at 27 weeks and four days of gestational age, and he weighed 1180 g. He was born to a 32-year-old gravida 7, para 3, abortion 3, a known case of dextrocardia, via emergency cesarean section due to preterm premature rupture of the membranes (PPROM) for 33 h. The antenatal follow-up results were unremarkable. The Apgar scores were 6 at 1 min, 8 at 5 min, and 9 at 10 min.

**Figure 1:**
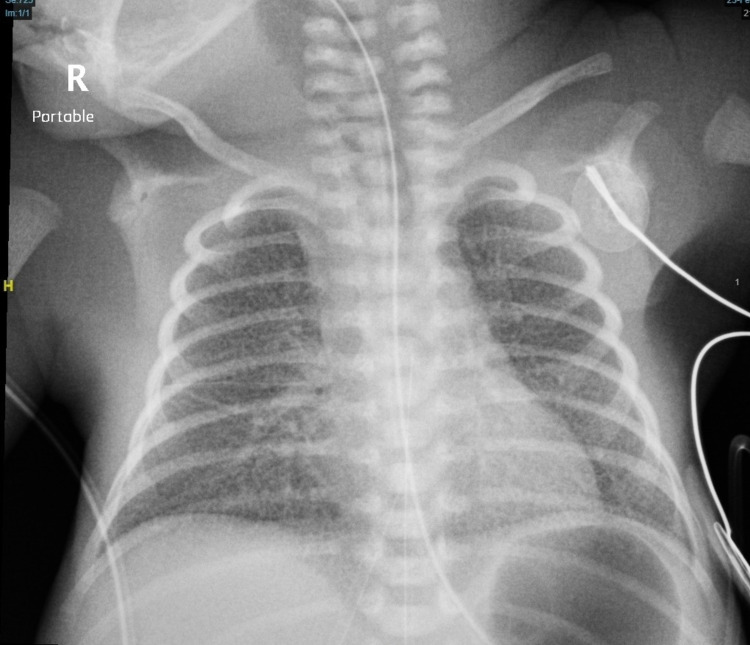
Chest radiography revealed bilateral diffused granular infiltration.

The golden hour measures were applied, and treatment with continuous positive airway pressure (CPAP) was started. Then, the baby was transferred to the neonatal intensive care unit due to respiratory distress. Chest radiography revealed bilateral diffused granular infiltration (Figure 1).

The initial complete blood count, C-reactive protein, and electrolyte levels were normal. Moreover, blood culture was performed, and antibiotic therapy was then started. The baby had a stable respiratory status with acceptable oxygen saturation (SpO2) and other blood gas parameter levels for his gestational and postnatal ages. Therefore, CPAP was reduced gradually.

**Figure 2:**
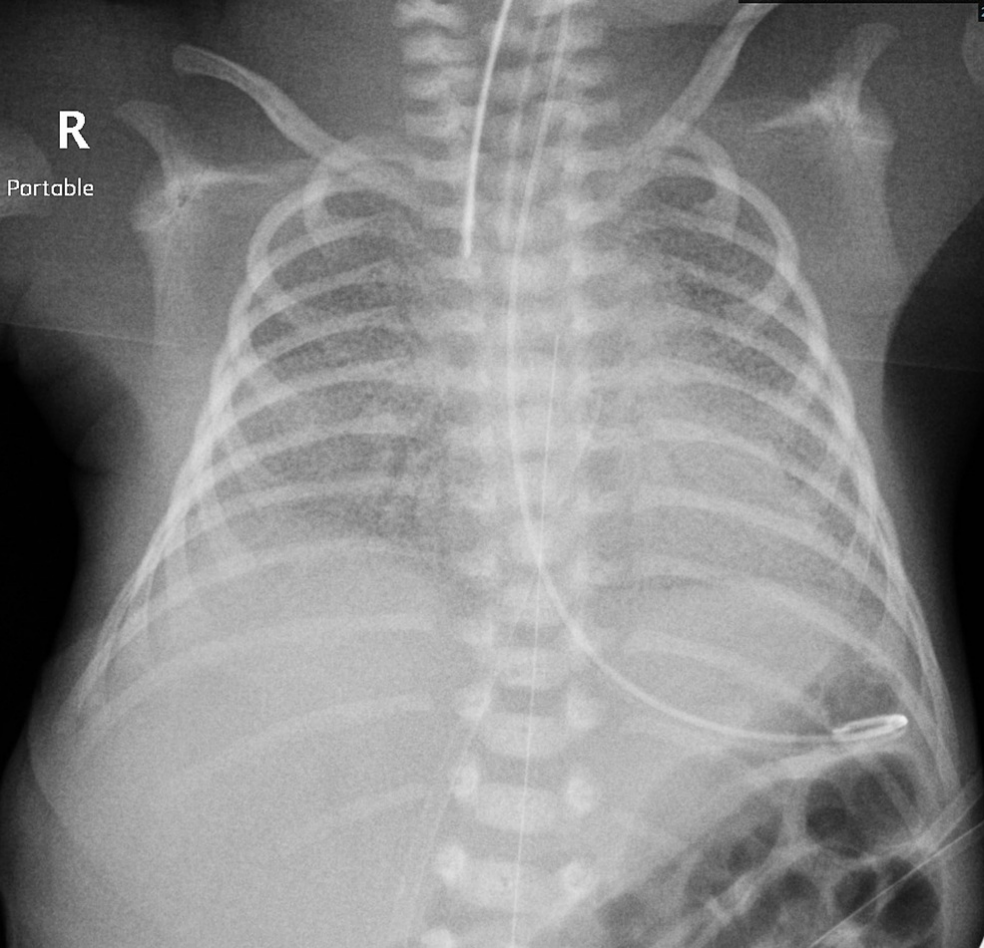
Chest radiography showed worsening respiratory distress syndrome but no pneumothorax.

At 13 hours of age, the baby started to desaturate, which required a higher level of nasal continuous positive airway pressure (NCPAP) support. Then, the patient developed apnea. Hence, he needed intubation, and the first dose of surfactant was administered. However, the baby’s SpO2 level was not maintained while on maximum conventional ventilation support. Therefore, the respiratory support was switched to high-frequency oscillatory ventilation. Despite the latter treatment, the baby continued to desaturate. Repeat chest radiography showed worsening respiratory distress syndrome but no pneumothorax (Figure 2).

Then, the baby received two extra doses of surfactant. Based on the presence of hypoxic respiratory failure, PAH was suspected clinically, and inotropic support was started.

The echocardiographic assessment showed a large patent ductus arteriosus with severe PAH and mild dilation of the right ventricle in addition to mild globally impaired ventricular functions. Therefore, the baby was treated with inhaled nitric oxide.

**Figure 3:**
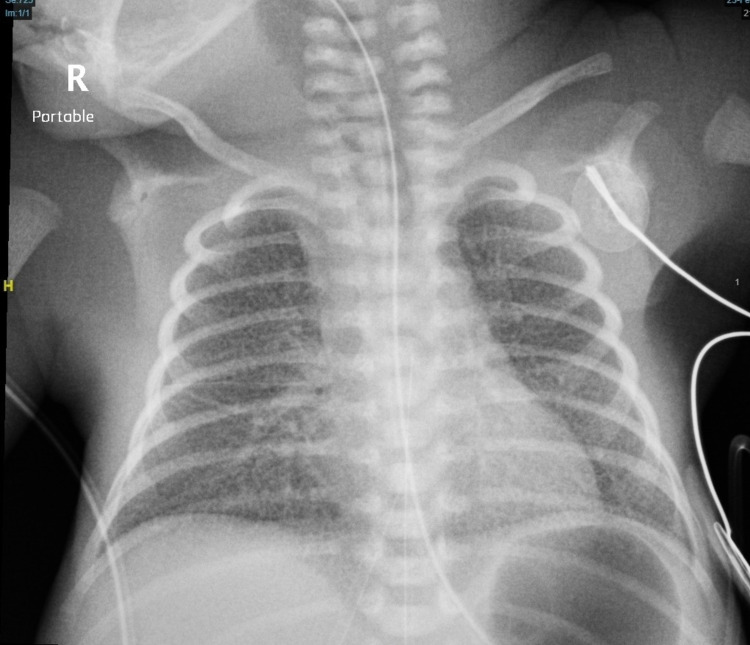
Chest radiography revealed better expansion of both lung fields compared with a previous study. However, bilateral diffuse granular infiltrate was noted.

His condition continued to deteriorate with a better expansion of both lung fields (Figure 3).

**Figure 4:**
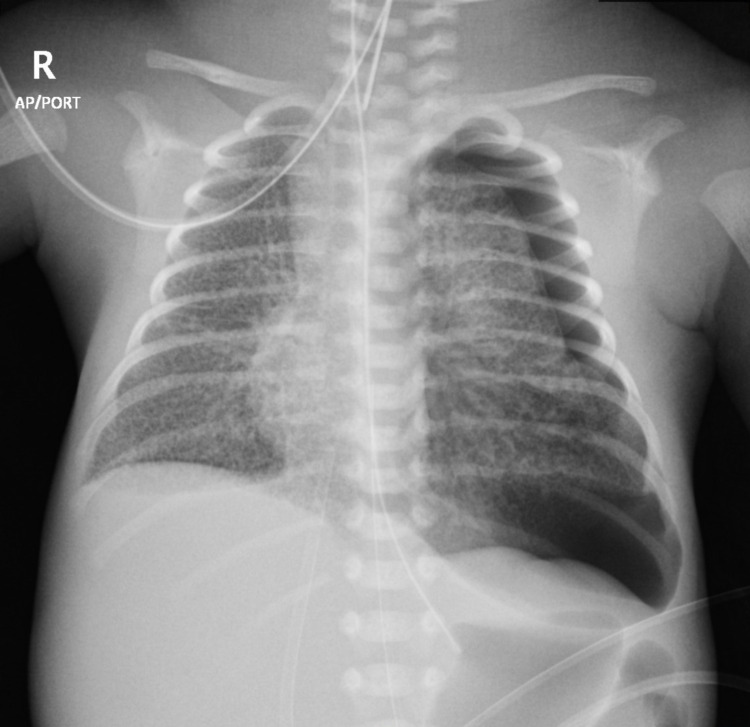
Chest radiography revealed newly developed left-sided pneumothorax.

**Figure 5:**
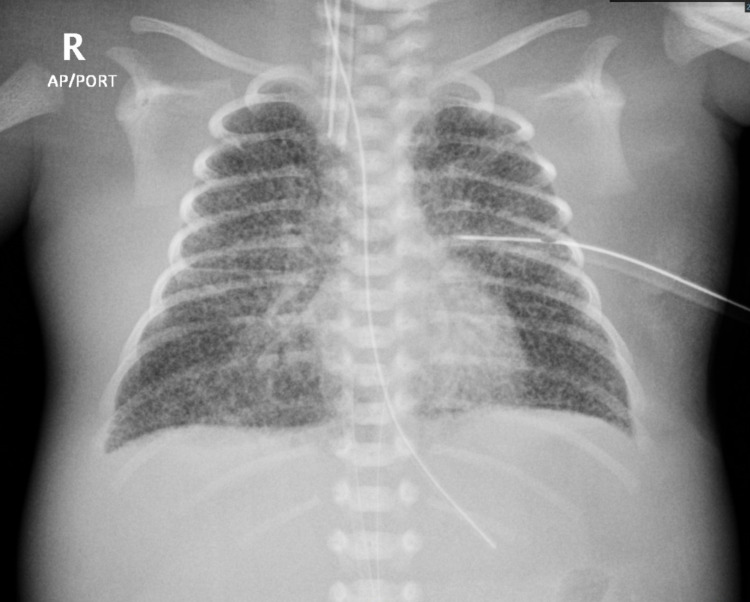
Chest radiography showed resolving pneumothorax after the insertion of left chest tube with re-expansion of the partially collapsed left lung.

He then developed left-sided tension pneumothorax, which required urgent intervention via chest tube insertion (Figures 4, 5).

The culture results remained negative. However, the C-reactive protein level slightly increased post-admission. Since the baby’s condition worsened, broad-spectrum antibiotics were administered.

Based on the clinical, laboratory, and echo findings and radiological features, we sent WES, which showed the homozygous *NF1* (NM_000267.3) variant (c.7127-1=/G>T P).

Despite maximum respiratory and hemodynamic support, the baby did not respond to treatment, and he died four days after birth.

## Discussion

NF1 is an autosomal dominant disease with approximately 100% transmission and 50% sporadic mutations. It is characterized by a risk of developing tumors in the ectodermal and mesodermal tissues [1,6,7]. Few studies showed that patients with NF1 had a higher risk of cardiac malformations. Nevertheless, the repetition and mechanism of these manifestations are still unknown [8,9]. The condition is diagnosed based on the presence of two or more of the following conditions according to the National Institutes of Health diagnostic criteria: café au lait macules, neurofibromas, axillary/inguinal freckling, Lisch nodules, distinctive osseous lesions, glioma, and/or family history of NF [10].

PAH is an extremely rare complication of NF1. Children with fatal complications such as cerebral infarction, fatal brain tumors, and hemorrhage may die before diagnosis [9]. The association between NF1 and PAH remains unclear. NF is a cause of systemic vasculopathy. This finding increases the strength of some theories showing that pulmonary vasculopathy is the mechanism underlying this correlation [8,11,12]. Further, the presence of neurofibrin, which is an NF1-encoded protein that plays a role in cell growth regulation and proliferation, supports the theory of pulmonary vasculopathy [13].

NF1 can be diagnosed via imaging with ventilation-perfusion scans and a high-resolution CT scan of the chest. These methods can identify bilateral filling defects and a mosaic pattern in the lungs, representing irregular perfusion [14]. There are no clear data nor recommendations for the use of approved drugs for group 5 PAH. However, despite the risk of pulmonary venous involvement in this group, vasodilators are considered effective [15].

Thus far, there is no research confirming whether NF1 is the primary cause of PAH in the neonatal age group. However, in the current case, a CT scan of the chest and biopsy were not performed because the baby was not stable.

## Conclusions

PAH is a rare fatal complication of NF1, and most cases in the literature involve adults. This case study aimed to present the possible etiology of PAH, and the results can be used as a basis for future research. Although few studies showed a higher incidence of cardiac malformation in NF1, 50% of children are born to healthy parents. However, further investigations should be performed to identify the risk of fatal complications in NF1, particularly among neonates.
